# Thermal Analysis Applied to Verapamil Hydrochloride Characterization in Pharmaceutical Formulations

**DOI:** 10.3390/molecules15042439

**Published:** 2010-04-08

**Authors:** Maria Irene Yoshida, Elionai Cassiana Lima Gomes, Cristina Duarte Vianna Soares, Alexandre Frinhani Cunha, Marcelo Antonio Oliveira

**Affiliations:** 1 Departamento de Química, Universidade Federal de Minas Gerais, Av. Pres. Antônio Carlos, 6627-31270-901 Belo Horizonte, MG, Brazil; 2 Departamento de Produtos Farmacêuticos, Universidade Federal de Minas Gerais, Av. Pres. Antônio Carlos, 6627-31270-901 Belo Horizonte, MG, Brazil; 3 Departamento de Ciências da Saúde, Biológicas e Agrárias, CEUNES, Universidade Federal do Espírito Santo, Rodovia BR 101 Norte, km 60, 29932-540 São Mateus, ES, Brazil

**Keywords:** verapamil hydrochloride, thermal analysis, degradation kinetics, characterization

## Abstract

Thermogravimetry (TG) and differential scanning calorimetry (DSC) are useful techniques that have been successfully applied in the pharmaceutical industry to reveal important information regarding the physicochemical properties of drug and excipient molecules such as polymorphism, stability, purity, formulation compatibility among others. Verapamil hydrochloride shows thermal stability up to 180 °C and melts at 146 °C, followed by total degradation. The drug is compatible with all the excipients evaluated. The drug showed degradation when subjected to oxidizing conditions, suggesting that the degradation product is 3,4-dimethoxybenzoic acid derived from alkyl side chain oxidation. Verapamil hydrochloride does not present the phenomenon of polymorphism under the conditions evaluated. Assessing the drug degradation kinetics, the drug had a shelf life (t_90_) of 56.7 years and a pharmaceutical formulation showed t_90_ of 6.8 years showing their high stability.

## 1. Introduction

Several reports in the literature demonstrate the importance of thermal analysis by thermogravimetry (TG) and differential scanning calorimetry (DSC) in the characterization, polymorphism identification, purity evaluation of drugs, compatibility studies for the pharmaceutical formulation, stability and drugs thermal decomposition [1,2,3,4,5,6,7,8,9,10,11,12].

Verapamil hydrochloride (VRP, C_27_H_38_N_2_O_4_·HCl, **I**), a phenylalkylamine calcium-channel blocker, has broadly been used as an anti-arrhythmic drug to manage supraventricular tachyarrhythmias. Due its vasodilating and negative inotropic properties, it has been indicated for the treatment of hypertension, ischemic heart disease, and hypertrophic cardiomyopathy. After oral administration of VRP to humans, the drug is rapidly absorbed and widely distributed. It undergoes an extensive hepatic and intestinal first-pass metabolism, resulting in a low extent of absolute oral bioavailability in humans [13,14]. Verapamil hydrochloride has also been described as an inhibitor of P-glycoprotein able to improve the chemotherapy response by reducing the resistance of cancer cells against antineoplasic agents [15].

Compound **I** is a crystalline powder that melts (with decomposition) between 138.5 and 140.5 °C and pKa 8.6. It shows UV absorption maxima at 232 and 278 nm in water. It is freely soluble in ethanol and methanol( >100 mg/mL); soluble in water (83 mg/mL); and practically insoluble in hexane (0.001 mg/mL). Verapamil hydrochloride has a partition coefficient, Log P (octanol/water) of 3.8 [16,17]. The drug degrades in methanol solution under UV light 254 nm, with 52% loss of activity after 2 h of exposure [18].

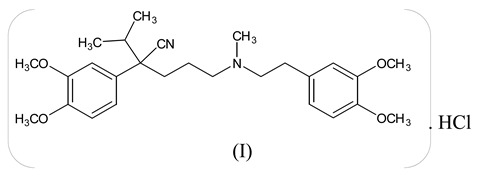



Verapamil hydrochloride is a low solubility and high permeability drug, class II, according to the biopharmaceutics classification system (BCS) [17,19] whereby the dissolution process is a rate-limiting step for the absorption and IVIVC (*in vivo-in vitro* correlation) can be expected [20]. Hence, it is important to evaluate drug features, such as the presence of polymorphism, stability and compatibility of the pharmaceutical formulation, as long as any change can directly influence its bioavailability.

Therefore, the aim of this study was to evaluate the thermal characterization of verapamil hydrochloride using a variety of techniques including TG, DSC, Fourier transform infrared spectroscopy (FTIR), liquid chromatography, and X-ray diffraction (XRD). The search of polymorphism and degradation products, together with formulation compatibility studies and thermal degradation kinetics, were carried out to help understanding the solid-state characterization, consequently, evaluate the quality control and stability for this important active pharmaceutical ingredient.

## 2. Results and Discussion

**Figure 1:**
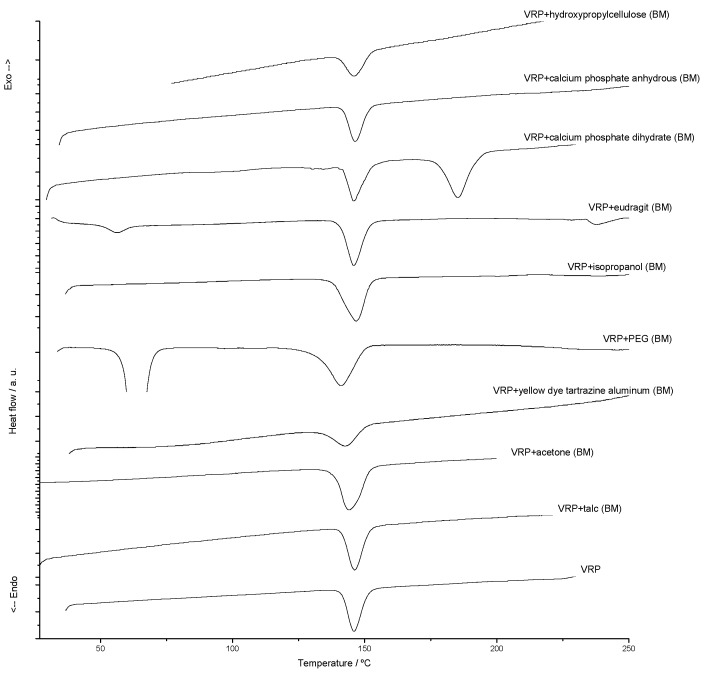
DSC curves for verapamil hydrochloride and its binary mixtures (BM, 1:1).

In the TG and DSC drug characterization, verapamil hydrochloride (Figure 1 bottom; Figure 6, respectively) showed thermal stability up to 180 °C, melting at 146 °C with an endothermic characteristic and fusion heat of *ΔH* = 121.9 J/g. After fusion, the DSC curve indicated the initiation of an exothermic process with positive slope, resulting in a complete degradation of the drug in two steps. The infrared spectrum showed no change compared to the reference spectrum, with main peaks at 1510, 1253, 1026, 1232, 1145 and 1587 cm^-1^ [17,21].

In compatibility studies, thermal analysis techniques allow the prior choice of more stable pharmaceutical formulations at a very short time, by means of evaluation of interactions that may exist, first, in their binary mixtures, and later in multicomponent mixtures. The quality of the provided information along with the speed of analysis is desirable for the pharmaceutical industry, but do not replace the conventional stability studies implied by law [22]. The DSC curves applied to compatibility studies may show changes in the fusion range, shape or area of the peaks and appearance and disappearance of thermal events after mixing two components, indicating interactions or chemical reactions, which must be confirmed by other analytical techniques.

By assessing the formulation compatibility using binary mixtures (Figure 1), changes can be seen in the fusion enthalpy value of verapamil hydrochloride in the heat enthalpy *ΔH* (J/g). However, the fusion range of the drug in the binary mixtures remained the same. The most significant thermal event occurred at the same temperature range, with small changes related to the binary mixture, not characterizing interactions [23,24]. The liquid ingredients of pharmaceutical formulations, which are acetone, ethanol and isopropanol, were compatible with verapamil hydrochloride.

**Figure 2:**
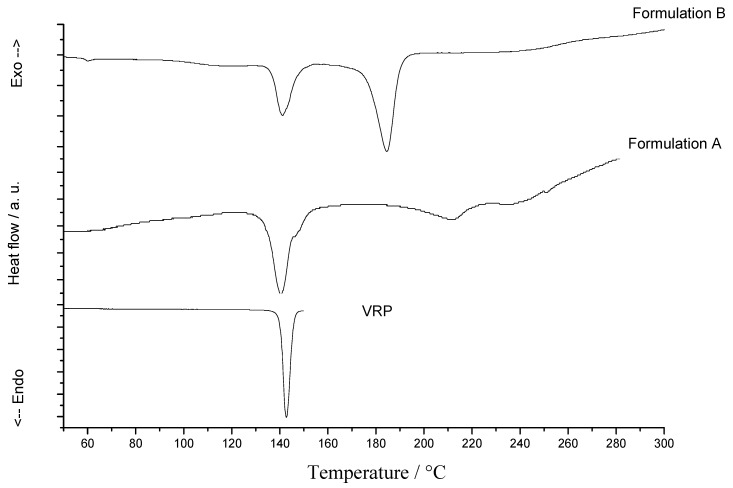
DSC curves of verapamil hydrochloride (VRP) from two local market formulations A and B.

In the multicomponent mixtures assessments (Figure 2), the overlap of DSC curves of drug and formulations A and B shows the drug fusion, demonstrating the compatibility of these formulations. Nunes and collaborators reported on the verapamil hydrochloride compatibility with common excipients used in tablet formulation by means of studies on binary mixtures (drug-excipients) and degradation kinetics using the non-isothermal method of Ozawa [25]. 

A HPLC/UV-DAD method was validated for verapamil hydrochloride in the presence of degradation products. A retention factor (k’) of 1.72, peak symmetry (As) of 1.05, theoretical plates/column (N) of 2556, repeatability and intermediate precision (RSD less than 1%), intra-day and inter-days accuracy with percentage recovery of 99.78% and 101.62% respectively, were satisfactorily obtained. Linear correlation coefficient (r) was greater than 0.99 in the range of 1 to 60 μg/mL. Detection limit of 0.18 μg/mL and quantification limit of 0.55 μg/mL. Selectivity studies, performed after drug stress conditions, and robustness were appropriate. 

**Figure 3:**
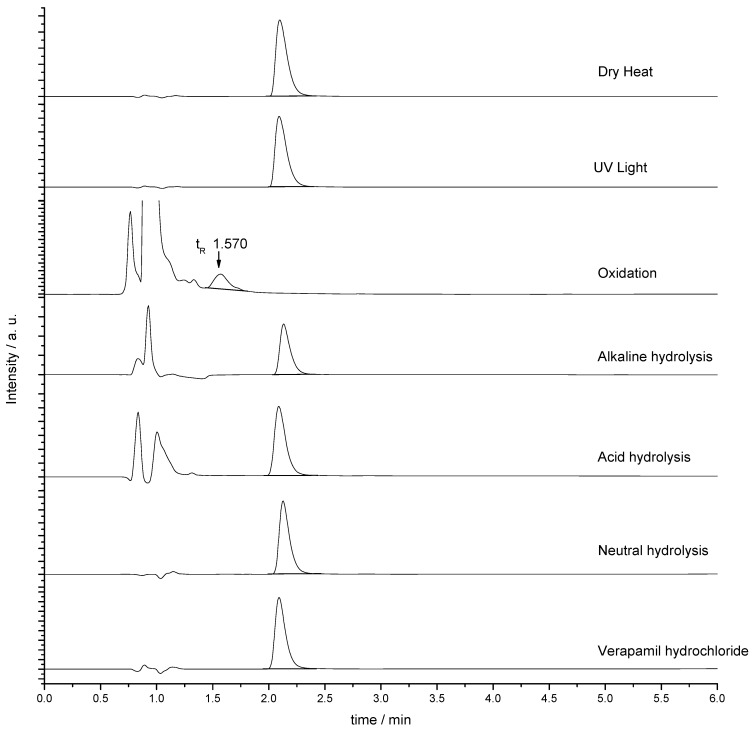
Verapamil hydrochloride chromatogram before (bottom) and after application of diverse stress conditions: neutral hydrolysis; acid hydrolysis; basic hydrolysis; oxidation; exposure to UV light; and exposure to temperature (dry heat).

The chromatograms of verapamil hydrochloride obtained before and after exposure to each stress conditions can be seen in Figure 3. Data show that degradation was mostly due to oxidation, and the peak t_R_ (retention time) 0.916 min refers to the hydrogen peroxide peak.

**Figure 4:**
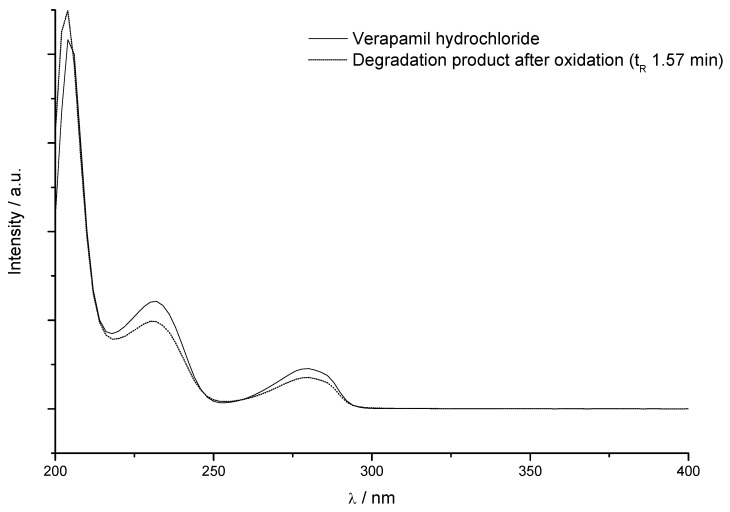
Overlay of UV spectra of verapamil hydrochloride (t_R_ 2.09, k’ 1.72) before (^___^) and after stress conditions (---) obtained for degraded solutions under oxidation (t_R_ 1.570, k’ 1.04).

The spectra of the degradation product peak after oxidation was compared with verapamil hydrochloride spectra (Figure 4), using HPLC with UV/DAD detector. The degradation product at t_R_ 1.57 min presented a similarity index (SI) of 0.9982, what indicates a very similar chromophore structure. When the retention time of the analyte peak is located as close as possible to the retention time of the reference peak and both of them have spectra with SI greater than 0.99, the peaks refer to similar compounds [17].

The aromatic compounds oxidation occurs due to oxidation of the side chains of alkyl groups. Given the similarity of the UV spectra of verapamil hydrochloride with the degradation product at t_R_ 1.5 min, suggests that the degradation product is 3,4-dimethoxybenzoic acid (C_9_H_10_O_4_). This compound has a more polar molecular structure compared to verapamil hydrochloride, what explains its lower retention in reverse phase column, which possess non-polar characteristics.

The attempt at identification of verapamil hydrochloride polymorphism began with the search by DSC analysis at different temperature rates. Heating rates of 2 and 20 °C/min under nitrogen atmosphere, from room temperature up to 180 °C showed no crystalline transition events and no double melting peaks, which rules out the presence of polymorphs in verapamil hydrochloride.

Recrystallization was performed under different conditions, such as different solvents, temperatures and solution saturations. There was no detection of formation of different crystalline forms after evaluation of the crystals by XRD and DSC. By optical microscopy, the crystals are prismatic after observation.

**Figure 5:**
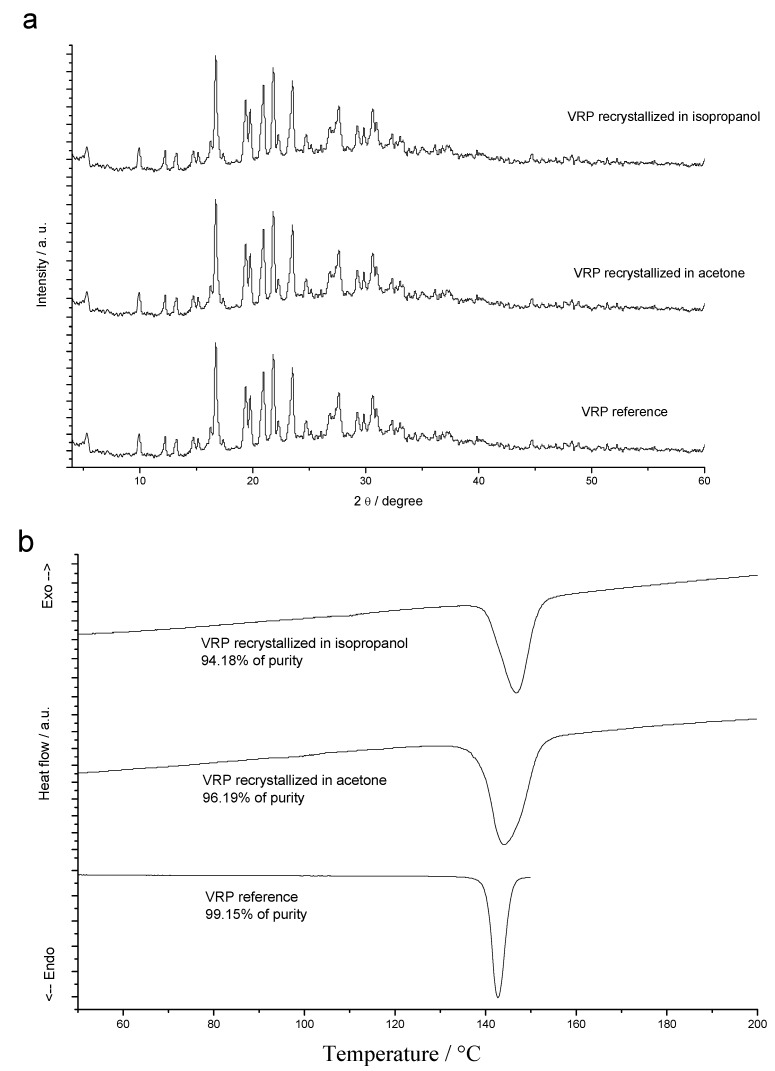
(a) XRD and (b) DSC results obtained for verapamil hydrochloride (VRP): reference, recrystallized in acetone and in isopropanol.

In XRD analysis, no difference of crystallinity between the crystals of the drug before (pattern) and after recrystallization in acetone or isopropanol (Figure 5a) was observed on the angle and intensity. This suggests the same crystal unit cell and crystal habit. In DSC curves (Figure 5b), there is a widening of fusion peak of the crystals obtained after recrystallization indicating that impurities may be present in the process. The verapamil hydrochloride pattern showed a more symmetrical and fine endotherm. Using the Van't Hoff equation, a peak purity of 99.15% for verapamil hydrochloride pattern was obtained, 96.19% and 94.18% of purity for crystals from acetone and from isopropanol respectively. However, different crystalline transition events and double melting peaks, what can be indicative of polymorphism, were not observed. The results suggest the formation of crystals with low purity, due to enlargement of the peak and lowering of T_onset_.

**Figure 6:**
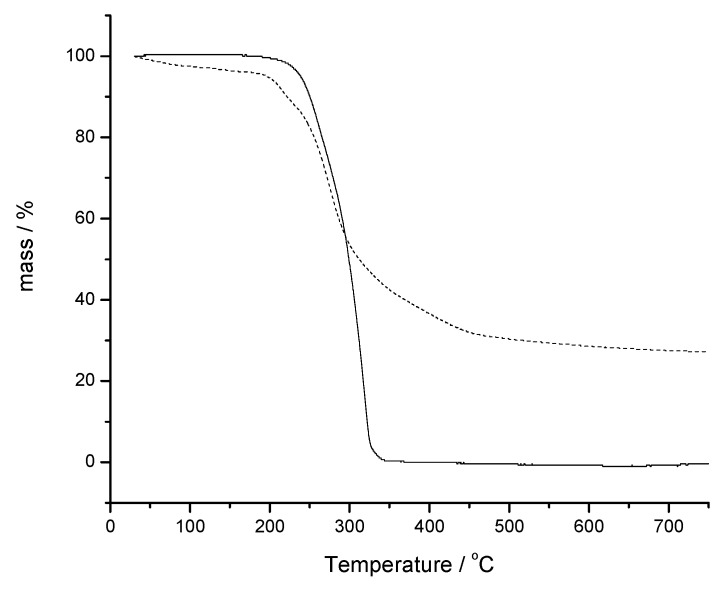
TG curves. Verapamil hydrochloride (^___^); and pharmaceutical formulation (---) containing verapamil hydrochloride.

The isothermal degradation kinetics was performed to assess the stability of the drug and pharmaceutical formulation as well as to predict the shelf life at 25 °C. Figure 6 shows the dynamic TG curves of the drug and pharmaceutical formulation in which can be seen the start of degradation in the same temperature range.

Isothermal TG curves were performed at the initial stage of degradation at temperatures of 190 to 240 °C, and curve fits in the zero order model for the drug, and second order model for the pharmaceutical formulation were obtained. Table 1 shows the values of correlation coefficient (r) and the rate constants (*k*) for the adjustments.

The kinetic data for the drug and pharmaceutical formulation were carried out according to zero order model, which in verapamil hydrochloride presented a better fit. The activation energy (Ea), the rate constants, and the shelf life at 25 °C were calculated by isothermal degradation kinetics.

**Figure 7:**
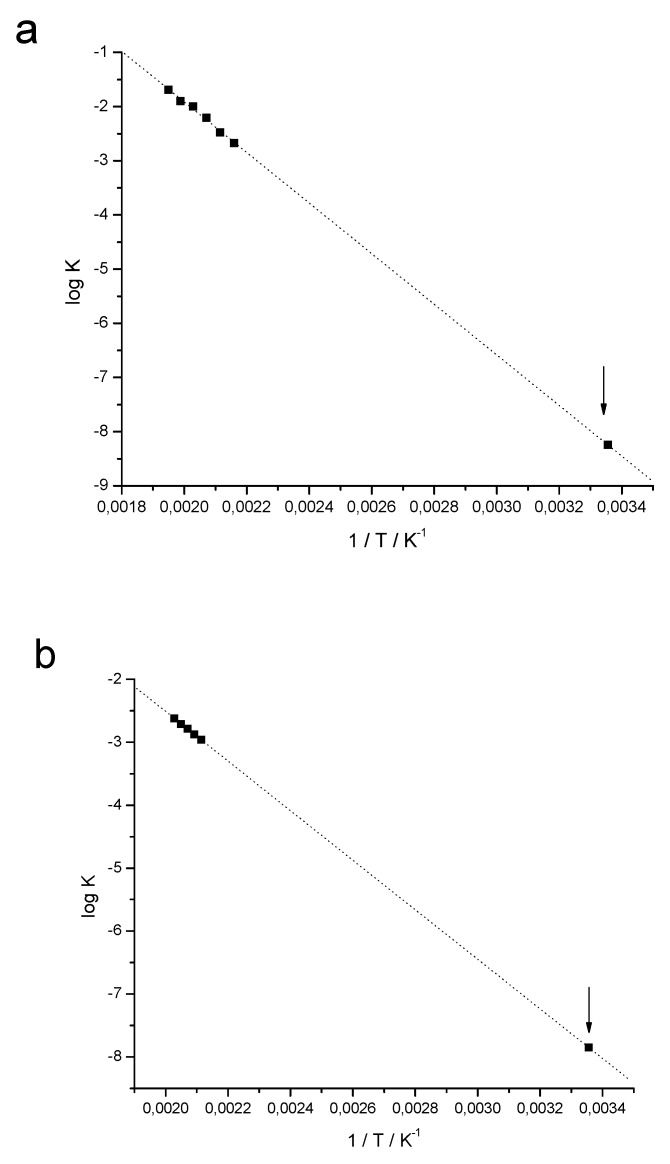
Graphic of the Arrhenius equation with values extrapolated to 25 °C for (a) verapamil hydrochloride, and for (b) pharmaceutical formulation.

Figure 7 shows the graph on Arrhenius equation, 1/T *vs.* log *k*. The slope of the line is defined by Ea/(2.303 × R), where the activation energy can be calculated by multiplying slope value by gas constant R (8,314 J·mol^-1^·K^-1^) and by 2.303. The linear regression calculated for the kinetic data of the drug led to Equation (1), with correlation coefficient of 0.9956 (r). The activation energy calculated for the verapamil hydrochloride was 89.4 kJ·mol^-1^:

log *k* = -4668.7 × 1/T + 7.4204 (1)

For the pharmaceutical formulation, the linear regression calculated using kinetic data show Equation (2), with correlation coefficient of 0.9992 (r). The activation energy calculated for the formulation was 75.4 kJ·mol^-1^:

log *k* = -3938.0 × 1/T + 5.3648 (2)

It was possible to calculate the rate constant of reaction (*k*) at 25 °C by extrapolation (highlighted, Figure 7) for the drug and for pharmaceutical formulation, as follows:

*k*_25_ drug = 5.67284 × 10^-9^ s^-1^

*k*_25_ pharmaceutical formulation = 1.41244 × 10^-8^ s^-1^

Given *k*_25 °C_ value, the shef life, t_90_, was calculated according to Equation (3) at 25 °C, whereas the degradation follows the zero order model. The concentration of verapamil hydrochloride in the tablet formulation was considered 30% as Co (initial concentration), since the average weight of the tablets were around 270 mg. A value of 56.7 years of shelf life was obtained for the drug, and 6.8 years for the pharmaceutical formulation. A great value for formulation expiration date is justified by the absence of incompatibility with the excipients evaluated. The Ea (activation energy) was 89.4 kJ·mol^-1^ and 74.4 kJ·mol^-1^ for drug and for pharmaceutical formulation, respectively demonstrating their stability. It can be observed in Table 7 that the degradation rate (*k*) is always higher in the pharmaceutical formulation when compared to the drug, at each temperature isotherm.

t_90_ = (0,1 × Co)/*k* (3)

where *k* = rate constant (s^-1^), Co = initial concentration (%).

## 3. Experimental

### 3.1. General

Verapamil hydrochloride characterization was performed using TG, DSC and IR. TG curves (TGA50H Shimadzu thermobalance). Conditions used were heating rate 10 °C/min, from room temperature up to 750 °C, nitrogen flow rate 50 mL/min, with a mass of 5.0 mg in an alumina crucible. DSC curves (DSC50 Shimadzu calorimeter) were obtained under nitrogen flow rate 50 mL/min, heating rate 10 °C/min from room temperature up to 400 °C. The aluminum crucible was partially closed with about 0.5 mg of sample. The assessment of purity by DSC was made by Van't Hoff equation using the Shimadzu Purity Determination Program Software, version 2.20. The DSC and TG temperature axes were calibrated with indium (99.99%; melting point, 156.60 °C) and by the Curie point of Ni (353 °C), respectively, heated at the same rates used for the samples.

IR experiments were carried out using a Perkin Elmer Spectrum One spectrometer by means of dispersion KBr disks.

Compatibility studies were performed by DSC technique, considering two local market formulations containing 80 mg of verapamil hydrochloride, coated tablets: a generic (A) and a reference (B); and a simulated (C) pharmaceutical formulation presented in the *Handbook of pharmaceutical manufacturing formulations: compressed solid products* [26] were evaluated. The excipients of each formulation were (i) A - acetone, ethyl alcohol, isopropyl alcohol, corn starch, tartrazine yellow dye, titanium dioxide, magnesium stearate, Eudragit E-100, Croscarmellose, calcium phosphate anhydrous, Povidone 30 (Kollidon k30), lactose M-100, polyethylene glycol 6000, colloidal silicon dioxide and talc; (ii) B - microcrystalline cellulose, croscarmellose sodium, colloidal silicon dioxide, magnesium stearate, calcium phosphate dihydrate, hydroxypropylmethylcellusose, sodium lauryl sulphate, polyethylene glycol 6000, talc and titanium dioxide; (iii) C - Ludipress ® (93% alpha lactose monohydrate, polyvinylpyrrolidone 3.5%, and 3.5% polyvinylpyrrolidone crosslinked), magnesium magnesium stearate and Aerosil 200 [26].

All ingredients listed for the tablets development were individually evaluated by DSC. In addition, a 1:1 ratio binary mixtures of drug to each excipient of the commercial pharmaceutical formulations, and placebo formulation (C) were tested in order to evaluate verapamil hydrochloride thermal behavior, as well as, pharmaceutical formulations compatibility. For the search for drug-excipient interactions in the binary mixtures 5 mg of drug were used with the same amount of excipient, in order to maximize the probability of observing an interaction. Then, multicomponent mixtures, as it occurs in dosage forms were evaluated [27,28,29].

Chromatographic studies using HPLC/UV-DAD (HP 1200, Agilent) were performed. The search for identifying degradation products after drug stress conditions could possibly be correlated with the degradation products from the incompatibilities found by thermoanalytical studies of DSC. The stress conditions (intrinsic stability) of verapamil hydrochloride were systematically investigated after 4 hours of exposure under distinct conditions: (i) dry heat at 105 °C, (ii) reflux over steam bath in water, (iii) in 1 M NaOH, (iv) in 1 M HCl, (v) in 3% H_2_O_2_, and (vi) UV light (254 nm). The chromatographic conditions used were: RP18 column (ODS, 250 × 4.6 mm, 5 μm, Merck), mobile phase: acetonitrile/acetic acid 0.3% v/v pH 4.9 (70:30), 2 mL/min, injection volume: 10 μL, UV-DAD detection λ 278 nm, 30 °C, samples concentration 40 μ g/mL in acetonitrile [21,30,31].

Drug recrystallization under different conditions was assessed using dichloromethane, methanol, ethanol, water, acetone or hexane; room (30 °C) or cooled (-10 °C) temperatures; saturated or diluted solutions. Analysis was performed by DSC, thermo-optical analysis (TOA) (FP90 and FP82OA Mettler Toledo), optical microscopy coupled with camera (Siedentopf), and X-ray powder diffraction (XRD). For the XRD experiments, a Geigerflex Rigaku diffractometer with cobalt tube (CoKα), operating voltage at 32.5 kV and 25.0 mA current was employed.

The stability evaluation of the drug and pharmaceutical formulation was performed by thermal analysis in order to determine shelf life at 25 °C by isotermic degradation kinetics. Dynamic TG curves and definition of the initial stage of degradation for drug and for pharmaceutical formulation were acquired. After, isotherms TG curves were carried in onset degradation temperatures for both, drug and pharmaceutical formulation, in order to assess the best fit of mathematical models for the isotherms at zero order (time *versus* % mass), first order (time *versus* log % mass) or second order (time versus 1/% mass). The mathematical model that provides the best linear correlation coefficient (r closer to 1.0000) represents the standard isothermal degradation. After establishing the reaction order, the reaction rate (*k*) at 25 °C was calculated by extrapolation using the Arrhenius equation. Using *k*_25 °C_ value, the shelf life, t_90_, were calculated for the drug and for pharmaceutical formulation. t_90_ represents the time interval required for the drug concentration reach 90% of the initial concentration value and it is accepted as the shelf life determination [10,23,32,33].

## 4. Conclusions

Verapamil hydrochloride showed thermal stability up to 180 °C and a melting point at 150 °C, followed by total degradation. The drug was compatible with all excipients evaluated. Verapamil hydrochloride showed degradation when subjected to oxidizing conditions, suggesting that the degradation product is the 3,4-dimethoxybenzoic acid, derived from the alkyl side chain oxidation. Verapamil hydrochloride did not present the phenomenon of polymorphism in the conditions evaluated. Assessing the degradation kinetic of the drug, the molecule showed a t_90_ of 56.7 years, and did not present problems of incompatibility. Pharmaceutical formulation presented a shelf-life, t_90,_ of 6.8 years.

## Figures and Tables

**Table:** **a**

Temperature / °C		ORDER		
	Parameter	zero	first	second
**190**	r	0.9991	0.9961	0.9910
	*k*	2.10 × 10^-03^	2.46 × 10^-05^	2.90 × 10^-07^
**200**	r	0.9999	0.9967	0.9869
	*k*	3.36 × 10^-03^	4.49 × 10^-05^	6.13 × 10^-07^
**210**	r	0.9997	0.9841	0.9265
	*k*	6.19 × 10^-03^	1.15 × 10^-04^	2.44 × 10^-06^
**220**	r	0.9998	0.9862	0.9436
	*k*	9.97 × 10^-03^	1.75 × 10^-04^	3.37 × 10^-06^
**230**	r	0.9955	0.9966	0.9665
	*k*	1.27 × 10^-02^	2.33 × 10^-04^	4.73 × 10^-06^
**240**	r	0.9887	0.9996	0.9802
	*k*	2.04 × 10^-02^	3.73 × 10^-04^	7.47 × 10^-06^

**Table:** **b**

Temperature / °C		ORDER		
	Parameter	zero	first	second
**200**	r	0.9560	0.9647	0.9722
	*k*	1.10 × 10^-03^	1.33 × 10^-05^	1.62 × 10^-07^
**205**	r	0.9544	0.9643	0.9727
	*k*	1.33 × 10^-03^	1.63 × 10^-05^	2.03 × 10^-07^
**210**	r	0.9602	0.9697	0.9777
	*k*	1.65 × 10^-03^	2.04 × 10^-05^	2.54 × 10^-07^
**215**	r	0.9582	0.9700	0.9796
	*k*	1.94 × 10^-03^	2.51 × 10^-05^	3.27 × 10^-07^
**220**	r	0.9579	0.9715	0.9822
	*k*	2.40 × 10^-03^	3.21 × 10^-05^	4.31 × 10^-07^
